# Long-term follow-up of a phase 2 study of oral teriflunomide in relapsing multiple sclerosis: safety and efficacy results up to 8.5 years

**DOI:** 10.1177/1352458512436594

**Published:** 2012-09

**Authors:** Christian Confavreux, David K Li, Mark S Freedman, Philippe Truffinet, Hadj Benzerdjeb, Dazhe Wang, Amit Bar-Or, Anthony L Traboulsee, Lucy E Reiman, Paul W O’Connor, for the Teriflunomide Multiple Sclerosis Trial Group

**Affiliations:** 1 Hôpital Neurologique, Université Claude Bernard Lyon 1, France; 2 University of British Columbia and MS/MRI Research Group, Canada; 3 University of Ottawa, Canada; 4 MS DPU, sanofi-aventis, France; 5 Global Pharmacovigilance and Epidemiology, sanofi-aventis, France; 6 Biostatistics and Programming, sanofi-aventis, USA; 7 McGill University, Canada; 8 Fishawack Communications Ltd, Abingdon, UK; 9 University of Toronto, Canada

**Keywords:** Multiple sclerosis, teriflunomide, oral therapy, long-term treatment, clinical trials

## Abstract

**Background:**

Teriflunomide, an oral disease-modifying therapy in development for patients with relapsing forms of multiple sclerosis (RMS), was well tolerated and effective in reducing magnetic resonance imaging (MRI) lesions in 179 RMS patients in a phase 2 36-week, placebo-controlled study.

**Methods:**

A total of 147 patients who completed the core study entered an open-label extension. Teriflunomide patients continued their assigned dose, and placebo patients were re-allocated to teriflunomide, 7 mg/day or 14 mg/day. An interim analysis was performed at a cut-off on January 8 2010.

**Results:**

The mean and median duration of study treatment, including both the core and extension phase, from baseline to the interim cut-off, was 5.6 years (standard deviation: 2.7 years) and 7.1 years (range: 0.05–8.5 years), respectively. Of 147 patients, 62 (42.2%) discontinued (19% due to treatment-emergent adverse events (TEAEs)). The most common TEAEs were mild infections, fatigue, sensory disturbances and diarrhoea. No serious opportunistic infections occurred, with no discontinuations due to infection. Asymptomatic alanine aminotransferase increases (≤3× upper limit of normal (ULN)) were common (7 mg, 64.2%; 14 mg, 62.1%); increases >3×ULN were similar across groups (7 mg, 12.3%; 14 mg, 12.1%). Mild decreases in neutrophil counts occurred; none led to discontinuation. The incidence of malignancies was comparable to that of the general population, and cases were not reminiscent of those observed in immunocompromised patients. Annualised relapse rates remained low, minimal disability progression was observed, with a dose-dependent benefit with teriflunomide 14 mg for several MRI parameters.

**Conclusion:**

Teriflunomide had a favourable safety profile for up to 8.5 years.

## Introduction

Teriflunomide is a novel oral disease-modifying therapy (DMT) being investigated for the treatment of relapsing forms of multiple sclerosis (RMS).^1^ Teriflunomide selectively and reversibly inhibits the mitochondrial enzyme dihydroorotate dehydrogenase, required for de novo pyrimidine synthesis. As a consequence, teriflunomide blocks the activation and proliferation of stimulated lymphocytes such as T- and B-cells, which require de novo synthesis of pyrimidine to expand. Slowly dividing or resting cells, which rely on the salvage pathway for pyrimidine synthesis, are relatively unaffected by teriflunomide.^2–4^

Teriflunomide delayed the onset of disease and improved neurological findings in the Dark Agouti rat model of experimental autoimmune encephalomyelitis.^5,6^ In clinical trials, teriflunomide has been evaluated as two once-daily oral dosages, 7 mg and 14 mg, both as monotherapy and as an adjunct to existing DMTs.^7–10^ The pivotal phase 3 TEMSO (TEriflunomide Multiple Sclerosis Oral) trial demonstrated that teriflunomide monotherapy at both doses significantly reduced the annualised relapse rate (ARR) and significantly increased the time to sustained disability progression (12 weeks) at the higher dose.^10^

A previous 36-week placebo-controlled phase 2 study of 179 patients with RMS showed that teriflunomide monotherapy (7 mg or 14 mg) was well tolerated and reduced the number of combined unique active lesions by >61%.^9^ Here, we report the safety and efficacy results from an open-label, long-term extension of this study.

## Methods

### Study population

Patients with RMS aged 18–65 years, who completed the 36-week placebo-controlled core study, were eligible for inclusion in the extension.^9^ Eligible subjects in the core study had an Expanded Disability Status Scale (EDSS) score of ≤6 and at least two clinical relapses in the previous 3 years and one during the preceding year. Patients were excluded if they had clinically relevant cardiovascular, hepatic, endocrine or other major systemic disease. Female patients were required to use effective contraception throughout the trial and until the end of a rapid drug elimination procedure (or alternatively to continue for 24 months after drug discontinuation).^11^ Concomitant treatment with immunosuppressive, immunomodulatory, or other DMTs was not permitted.

### Study design

Patients were initially randomised (1:1:1) to receive a once-daily oral dose of teriflunomide 7 mg or 14 mg, or placebo, for 36 weeks. Randomisation was stratified by a baseline EDSS score (≤3.5 and ≥4.0). Following completion of the core study, patients who were originally randomised to teriflunomide continued their assigned treatment and those receiving the placebo were re-allocated to teriflunomide, 7 mg or 14 mg, according to the predefined randomisation schedule. At the time of reporting, the extension was still ongoing.

### Study objectives

The primary objective was to assess the long-term safety of teriflunomide in patients with RMS. Secondary objectives were to assess the long-term efficacy of teriflunomide in terms of relapse rate, disability accumulation, magnetic resonance imaging (MRI) outcomes and quality of life.

### Standard protocol approvals, registrations and patient consents

The study protocol was approved by an independent ethics committee, and in accordance with the Declaration of Helsinki, the International Conference on Harmonisation and Good Clinical Practice guidelines and local regulations. Enrolled patients provided written informed consent. ClinicalTrials.gov identifier: NCT00228163.

### Study procedures

#### Safety evaluations

Safety was assessed by monitoring treatment-emergent adverse events (TEAEs), vital signs and laboratory parameters throughout the core study and extension, with clinic visits every 12 weeks. TEAEs were defined as any adverse event (AE) that developed or worsened after the first dose of the study drug and up to 16 weeks following the last dose. Spontaneously reported AEs were recorded at clinic visits.

Blood samples to evaluate haematology for consistency and biochemistry were taken every 6 weeks. Serious hepatic disorders were defined as alanine aminotransferase (ALT)>8× the upper limit of normal (ULN) or any event with standard seriousness criteria. Vital signs (pulse, blood pressure), physical examinations, blood chemistry and urinalysis were assessed every 12 weeks.

#### Efficacy evaluations

The baseline was defined as the visit prior to the first dose of the study drug in the core study. Clinical efficacy outcomes (relapses, EDSS score) were assessed every 24 weeks. Analyses of ARR, the proportion of relapse-free patients and the time to first relapse were performed from the beginning of the extension. An increase in disability was defined as an increase in EDSS score of ≥1.0 in patients with a baseline EDSS score of ≤5.5 or an increase in EDSS score of ≥0.5 in patients with a baseline score of ≥5.5. MRI scans were performed every 48 weeks from the beginning of the extension and sent to the central reading centre (the MS/MRI Research Group at the University of British Columbia, Vancouver, Canada) for analysis. MRI scans were analysed for T2 lesion volume (or burden of disease (BOD), mm^3^) and cerebral volume as the predefined study endpoints. Cerebral volume was assessed using the brain parenchymal ratio (derived by subtracting the cerebrospinal fluid volume from the intradural volume and normalising to the whole intradural volume). Analyses of new and newly enlarging T2 lesions, gadolinium (Gd)-enhancing T1 lesions and newly active lesions (sum of Gd-enhancing T1 and non-enhancing new and newly enlarging T2 lesions to avoid double counting active lesions) were also performed. Multiple Sclerosis Quality of Life-54 (MSQoL-54) and Fatigue Impact Scale (FIS) instruments were performed every 24 weeks.

### Statistical analysis

Results are based on an interim analysis with an arbitrary cut-off of January 8 2010. The duration of teriflunomide exposure for each patient during both the core study and extension was evaluated.

Safety data (TEAEs) are presented for the core study and extension, for the patients who entered the extension and according to the treatment received during the extension only.

In accordance with the statistical analysis plan, no formal statistical tests were performed on the efficacy data. Efficacy evaluations were performed on the intention-to-treat (ITT) population, defined as all randomised patients who were exposed to the study drug in the extension. Efficacy data are presented as four treatment groups according to treatment allocation at randomisation and re-allocation at the start of the extension (i.e. placebo/7 mg, placebo/14 mg, 7 mg/7 mg or 14 mg/14 mg). For graphical presentation, efficacy data are presented irrespective of the treatment received during the core study as pooled 7-mg and 14-mg groups. Continuous data are summarised using the mean and standard deviation (SD), and categorical data by number and percentage. ARR was calculated as the ratio of the total number of relapses observed and the total patient-years of treatment during the extension.

## Results

### Subject disposition

**Figure 1.**
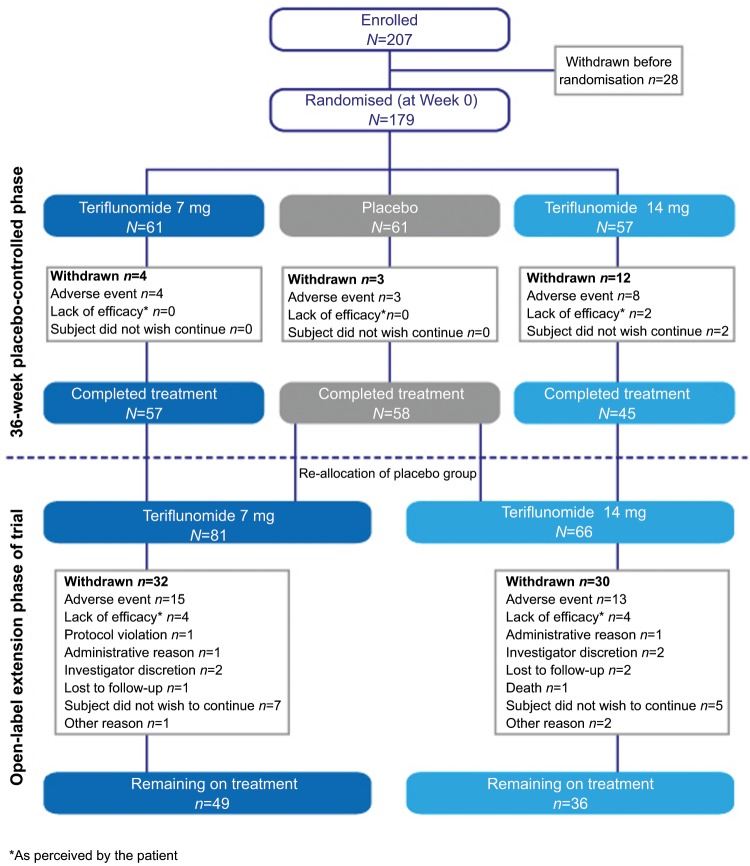
Subject disposition.

A total of 179 patients were randomised into the core study from April 26 2001 to March 17 2003, from 17 study centres across Canada and France. Of the 160 patients who completed the core study, 147 patients (92%) entered the extension. At the interim analysis cut-off, 62 (42.2%) patients had discontinued treatment, 28 (19%) due to TEAEs (Figure 1). The numbers and reasons for discontinuation were generally similar between dose groups and an even rate of discontinuations was observed throughout the study period. The mean and median duration of study treatment, including both the core and extension phase, from baseline to the interim cut-off, was 5.6 years (standard deviation: 2.7 years) and 7.1 years (ranging from 0.05 to 8.5 years), respectively.

### Demographics and disease characteristics

The majority of patients had relapsing–remitting MS, and a minority were diagnosed with secondary progressive MS (Table 1). Patients experienced a mean of 1.4 relapses during the year prior to the study, which was similar across treatment groups (Table 1). Patients in the 7-mg groups had a slightly longer duration since first onset of MS symptoms and higher mean EDSS scores, with slightly more patients with secondary progressive disease than those in the 14-mg groups.

**Table 1.** Patient demographics and clinical and MRI characteristics at baseline (start of the core study).

	Teriflunomide 7 mg			Teriflunomide 14 mg		
	Placebo/7 mg (*N*=29)	7 mg/7 mg (*N*=52)	Combined (*N*=81)	Placebo/14 mg (*N*=26)	14 mg/14 mg (*N*=40)	Combined (*N*=66)
**Baseline demographics**						
Age (years), mean (SD)	39.2 (8.1)	40.1 (9.7)	39.8 (9.1)	40.3 (9.9)	40.3 (9.9)	40.0 (9.4)
Sex (female), *n* (%)	19 (65.5)	39 (75.0)	58 (71.6)	32 (80.0)	32 (80.0)	64 (75.8)
Caucasian/white, *n* (%)	28 (96.6)	47 (90.4)	75 (92.6)	26 (100)	37 (92.5)	63 (95.5)
Country, *n* (%)						
France	2 (6.9)	5 (9.6)	7 (8.6)	2 (7.7)	4 (10.0)	6 (9.1)
Canada	27 (93.1)	47 (90.4)	74 (91.4)	24 (92.3)	36 (90.0)	60 (90.9)
**Clinical disease characteristics**						
Mean (SD) time since first symptoms of MS, years	7.9 (7.4)	10.7 (8.7)	9.7 (8.3)	9.3 (8.2)	8.5 (7.4)	8.8 (7.7)
Number of relapses within past 12 months						
Mean (SD)	1.5 (0.7)	1.4 (0.7)	1.4 (0.7)	1.4 (0.6)	1.5 (0.6)	1.4 (0.6)
Median (min:max)	1.0 (0.0:3.0)	1.0 (0.0:4.0)	1.0 (0.0:4.0)	1.0 (1.0:3.0)	1.0 (0.0:3.0)	1.0 (0.0:3.0)
MS subtype						
Relapsing–remitting, *n* (%)	24 (82.8)	46 (88.5)	70 (86.4)	24 (92.3)	37 (92.5)	61 (92.4)
Secondary progressive, *n* (%)	5 (17.2)	6 (11.5)	11 (13.6)	2 (7.7)	3 (7.5)	5 (7.6)
**EDSS**						
Mean (SD)	2.67 (1.67)	2.81 (1.54)	2.76 (1.58)	2.38 (1.39)	2.28 (1.62)	2.32 (1.52)
Median (min:max)	2.5 (0.0:6.0)	2.5 (0.0:6.0)	2.5 (0.0:6.0)	2.5 (0.0:5.5)	2.0 (0.0:6.0)	2.0 (0.0:6.0)
**MRI outcomes**						
**T2 burden of disease (mm^3^)**						
Mean (SD)	9504 (11214)	10716 (15058)	10282 (13744)	8794 (9630)	8300 (9765)	8495 (9641)
Median (min:max)	7118 (142:50073)	5562 (231:89046)	6247 (142:89046)	4631 (304:34985)	5713 (380:48261)	5320 (304:48261)
**Cerebral volume**						
Mean (SD)	0.80 (0.04)	0.79 (0.05)	0.79 (0.05)	0.80 (0.04)	0.79 (0.04)	0.80 (0.04)
Median (min:max)	0.81 (0.7:0.9)	0.80 (0.7:0.9)	0.80 (0.7:0.9)	0.80 (0.7:0.9)	0.79 (0.7:0.9)	0.80 (0.7:0.9)
**Gd-enhancing T1 lesions, *n***						
Mean (SD)	2.1 (4.4)	1.4 (2.8)	1.6 (3.4)	1.7 (3.6)	2.6 (6.6)	2.2 (5.6)
Median (min:max)	1.0 (0.0:18.0)	0.0 (0.0:14.0)	0.0 (0.0:18.0)	0.0 (0.0:14.0)	0.0 (0.0:35.0)	0.0 (0.0:35.0)

EDSS: Expanded Disability Status Scale; Gd: gadolinium; SD: standard deviation; MS: multiple sclerosis.

### Safety and tolerability

#### Overview of TEAEs and SAEs

Almost every patient reported at least one TEAE over the long study period (Table 2). The most commonly reported TEAEs were reported in the following categories and are described in more detail below: infections, hepatic disorders, gastrointestinal disorders, neurological disorders, psychiatric disorders and haematologic abnormalities (Table 2). Other specific AEs of interest included decreased hair density, blood pressure increase, hypersensitivity reaction, malignancies and pregnancies. Discontinuations due to TEAEs occurred for 15 (18.5%) and 13 (19.7%) patients in the 7-mg and 14-mg groups, respectively (Figure 1). Serious adverse events (SAEs) were reported in 29 (35.8%) and 19 (28.8%) patients in the 7-mg and 14-mg groups, respectively (Table 2); 11 (13.6%) and 9 (13.6%) patients discontinued due to serious TEAEs.

**Table 2.** Incidence (patients, *n*, %) of treatment-emergent adverse events throughout the study (≥20% crude incidence in either treatment group during the core study and the extension).

TEAE	Crude incidence, *n* (%)		Episodes of TEAEs per patient, N1 (N2/*n*)^a^	
	Teriflunomide 7 mg (*N*=81)	Teriflunomide 14 mg (*N*=66)	Teriflunomide 7 mg (*N*=81)	Teriflunomide 14 mg (*N*=66)
Any TEAE	80 (98.8)	66 (100)	41.7 (3338/80)	37.9 (2503/66)
**Infections and infestations**	**72 (88.9)**	**56 (84.8)**	**6.0 (431/72)**	**7.5 (421/56)**
Nasopharyngitis	37 (45.7)	35 (53.0)	2.6 (96/37)	1.9 (68/35)
Upper respiratory tract infection	29 (35.8)	29 (43.9)	2.2 (64/29)	2.2 (63/29)
Influenza	22 (27.2)	23 (34.8)	1.4 (31/22)	1.8 (41/23)
Urinary tract infection	19 (23.5)	15 (22.7)	2.8 (54/19)	2.9 (44/15)
**Gastrointestinal disorders**	**65 (80.2)**	**50 (75.8)**	**3.9 (253/65)**	**3.6 (180/50)**
Diarrhoea	22 (27.2)	25 (37.9)	1.6 (36/22)	1.6 (40/25)
Nausea	21 (25.9)	17 (25.8)	1.6 (34/21)	1.5 (25/17)
**Investigations**^a^	**59 (72.8)**	**53 (80.3)**	**4.8 (281/59)**	**5.5 (292/53)**
Alanine aminotransferase increased	24 (29.6)	19 (28.8)	1.9 (45/24)	2.1 (39/19)
Aspartate aminotransferase increased	10 (12.3)	15 (22.7)	1.9 (19/10)	1.5 (22/15)
**Nervous system disorders**	**77 (95.1)**	**61 (92.4)**	**10.5 (805/77)**	**8.5 (517/61)**
Hypoaesthesia	39 (48.1)	33 (50.0)	2.2 (86/39)	2.3 (77/33)
Headache	40 (49.4)	26 (39.4)	3.6 (144/40)	2.5 (64/26)
Pallanesthesia	27 (33.3)	20 (30.3)	1.7 (47/27)	1.3 (26/20)
Sensory disturbance	20 (24.7)	19 (28.8)	1.4 (28/20)	1.3 (24/19)
Dizziness	18 (22.2)	14 (21.2)	2.0 (36/18)	2.5 (35/14)
Hyperreflexia	19 (23.5)	14 (21.2)	1.7 (32/19)	1.9 (27/14)
Multiple sclerosis^b^	25 (30.9)	13 (19.7)	1.4 (36/25)	1.2 (15/13)
Coordination abnormal	20 (24.7)	10 (15.2)	1.5 (29/20)	1.4 (14/10)
**Psychiatric disorders**	**45 (55.6)**	**34 (51.5)**	**2.5 (113/45)**	**1.8 (61/34)**
Insomnia	28 (34.6)	17 (25.8)	1.4 (39/28)	1.3 (22/17)
Depression	20 (24.7)	10 (15.2)	1.4 (27/20)	1.6 (16/10)
**Skin and subcutaneous tissue disorders**	**51 (63.0)**	**42 (63.6)**	**2.7 (136/51)**	**2.6 (111/42)**
Hair thinning/decreased hair density^c^	17 (21.0)	18 (27.3)	1.5 (26/17)	1.4 (25/18)
Rash	18 (22.2)	11 (16.7)	1.2 (22/18)	1.9 (21/11)
**Musculoskeletal and connective tissue disorders**	**70 (86.4)**	**53 (80.3)**	**6.1 (424/70)**	**5.7 (301/53)**
Muscular weakness	34 (42.0)	25 (37.9)	2.6 (88/34)	2.2 (55/25)
Back pain	28 (34.6)	24 (36.4)	1.5 (41/28)	1.9 (46/24)
Pain in extremity	37 (45.7)	21 (31.8)	2.2 (83/37)	2.5 (52/21)
Paresthesia	25 (30.9)	20 (30.3)	1.9 (48/25)	1.9 (37/20)
Arthralgia	27 (33.3)	18 (27.3)	1.9 (51/27)	1.5 (27/18)
**General disorders**	**61 (75.3)**	**52 (78.8)**	**3.7 (228/61)**	**3.3 (169/52)**
Fatigue	39 (48.1)	32 (48.5)	1.9 (75/39)	2.2 (71/32)
Gait disturbance	17 (21.0)	11 (16.7)	2.2 (38/17)	1.2 (13/11)
Asthenia	17 (21.0)	8 (12.1)	1.3 (22/17)	1.1 (9/8)
**Renal and urinary disorders**	**33 (40.7)**	**23 (34.8)**	**2.5 (82/33)**	**2.6 (60/23)**
Micturition urgency	17 (21.0)	13 (19.7)	1.4 (24/17)	1.9 (25/13)
**Serious TEAEs**^d^				
Any serious TEAE	29 (35.8)	19 (28.8)	1.2 (35/29)	2.0 (38/19)
Hepatic enzyme increased	5 (6.2)	5 (7.6)	1.0 (5/5)	1.0 (5/5)
Alanine aminotransferase increased	1 (1.2)	2 (3.0)	1.0 (1/1)	1.5 (3/2)
Loss of consciousness	1 (1.2)	2 (3.0)	1.0 (1/1)	1.0 (2/2)
Neutropenia	0	2 (3.0)	0 (0/0)	1.5 (3/2)
Pneumonia	0	2 (3.0)	0 (0/0)	1.0 (2/2)
Multiple sclerosis^b^	3 (3.7)	0	1.0 (3/3)	0 (0/0)
Breast cancer	2 (2.5)	0	1.0 (2/2)	0 (0/0)

TEAE: treatment-emergent adverse event.

Data presented by Medical Dictionary for Regulatory Activities (MedDRA) preferred term and by decreasing order of frequency in the 14 mg dose group. The number (N1) represents the number of TEAEs per patient. It is calculated as the ratio of the total number of TEAEs (N2) and the total number of patients with at least one TEAE (*n*).

a Laboratory abnormalities reported as TEAEs were based on the investigator’s decision and/or on the following reporting thresholds (confirmed by a re-test): alanine aminotransferase ≥2x upper limit of normal (ULN) or bilirubin ≥2xULN; serum amylase or lipase levels ≥2xULN; neutrophil counts <1000 cells/μL.

b MS relapse, which was classed as a MS adverse event in the safety data set.

c MedDRA preferred term: Alopecia.

d More than 1 serious TEAE (crude incidence) reported in either treatment group.

One death was reported over the study period. A 51-year-old Caucasian female patient, who had been treated with teriflunomide 14 mg for 4.8 years, died from a sudden cardiac disorder a few hours after admission to hospital for ‘malaise’ with anxiety, hypotension and tachycardia. The patient had a medical history of dyspnoea, anxiety disorder, depression/delusions and Hashimoto’s thyroiditis with hypothyroidism. Additionally, 2 years earlier, the patient had experienced an SAE of respiratory failure in association with pneumonia and tachycardia. The patient was also concomitantly taking several medications, including propranolol, salmeterol, levothryrox, betamethasone, amantadine and oestradiol, and was given benzodiazepines at the time of the event. Therefore, underlying disease conditions and concomitant medications may have been contributory.

#### Number of TEAE episodes per patient

The number of episodes of any TEAE reported per patient over the study period was similar in both treatment groups (Table 2). Generally, for each individual TEAE, the number of episodes per patient was low and similar across teriflunomide groups (≤2.5 per patient). One exception was the number of episodes relating to infection (6.0 and 7.5 per patient for the 7-mg and 14-mg groups, respectively). Infections that occurred ≥2.5 times per patient included: nasopharyngitis, cystitis, urinary tract infection (Table 2) and oral herpes; there was a higher frequency per patient of oral herpes in the 14-mg group (2.8) than the 7-mg group (1.0). Other TEAEs that occurred ≥2.5 times per patient included: fall (1.4 and 2.7, for the 7-mg and 14-mg groups, respectively), muscular weakness (2.6 and 2.2), pain in extremity (2.2 and 2.5) and dizziness (2.0 and 2.5) (Table 2). A small number of TEAEs occurred ≥3.0 times per patient during the study, including headache (3.6 and 2.5, for the 7-mg and 14-mg groups, respectively), migraine (2.2 and 3.7), neutrophil count decrease (3.0 and 1.7) and white blood cell count decrease (5.0 and 1.6); all except migraine occurred less frequently in the 14-mg group.

The number of episodes of all types of SAE was low (≤1.5 per patient) and similar across both treatment groups (Table 2).

#### Infections

Infections were mainly of upper respiratory tract origin, including nasopharyngitis and upper respiratory tract infection. Influenza and urinary tract infection were also commonly reported (Table 2). As noted earlier, there was a higher incidence of oral herpes in the 14-mg group (12.1%) than in the 7-mg group (1.2%).

Isolated cases of appendicitis (one patient in the 7-mg group), bronchitis (one patient in the 7-mg group), pneumonia (two patients in the 14-mg group) and urinary tract infection (one patient in each group) were reported as SAEs. No progressive multifocal leukoencephalopathy or other serious opportunistic infections were reported, and there were no discontinuations due to infection.

#### Hepatic disorders

Asymptomatic ALT increases (≤3×ULN) were common, with similar incidence in the two treatment groups (64.2% and 62.1%, for 7 mg and 14 mg, respectively). The incidence of ALT increase >3×ULN was 12.3% and 12.1%, for 7 mg and 14 mg, respectively. The maximum ALT increase was <10×ULN. For those patients with significant increases, normalisation occurred for most cases within 2 months of treatment discontinuation. None of the ALT increases were symptomatic and no ALT >3×ULN was associated with total bilirubin >2×ULN. ALT >2×ULN could be reported as an AE by the investigator, resulting in a high frequency of TEAEs related to increased ALT (Table 2).

#### Gastrointestinal disorders

A higher proportion of patients reported diarrhoea in the 14-mg group compared with 7-mg group (Table 2). However, nausea, which was also commonly reported but was not associated with vomiting in the majority of cases, did not appear to have a dose-dependent effect. None of these TEAEs led to treatment discontinuation.

The proportion of patients with TEAEs potentially related to pancreatic disorders was 7.4% and 13.6% in the 7-mg and 14-mg groups, respectively. These were mainly asymptomatic increases (mostly <2×ULN) in pancreatic enzymes (including amylase and lipase), and none was serious or resulted in treatment discontinuation.

#### Neurological disorders

Commonly reported neurological TEAEs included hypoaesthesia, headache, pallanesthesia and sensory disturbance. Headaches were reported more frequently in the 7-mg group (Table 2). Fatigue, a common symptom of MS, was frequently reported in both treatment groups. Peripheral neuropathies (combining poly- and mono-neuropathies) were reported by 5 (6.2%) and 4 (6.1%) patients in the 7-mg and 14-mg groups, respectively; none was serious or led to treatment discontinuation.

#### Haematologic disorders

The reported TEAEs of asymptomatic laboratory findings related to white blood cell (WBC) counts included decreases in WBC counts (3.7% and 18.2%, in the 7-mg and 14-mg groups, respectively), neutrophil counts (7.4% and 19.7%) and lymphocyte counts (3.7% and 7.6%), with higher incidences in the 14-mg group. In a few cases, WBC disorder terminology was used to report the TEAE, including leukopenia (one patient in the 14 mg-group), lymphopenia (one patient in the 7-mg group and two patients in the 14-mg group) and neutropenia (two patients in the 7-mg group and three patients in the 14 mg-group). The effect size of decreases in WBC counts was low in all cases, and none led to treatment discontinuation.

#### Psychiatric disorders

The proportion of patients reporting insomnia and depression was lower in the 14-mg group compared with the 7-mg group (Table 2).

#### Decreased hair density

The proportion of patients reporting decreased hair density was higher in the 14-mg group compared with the 7-mg group (Table 2). Cases were mostly mild or moderate and transient in nature, and none was classed as an SAE or led to treatment discontinuation.

#### Blood pressure increase

An increase in the mean change from baseline was observed over time both for supine diastolic and systolic blood pressure. A total of 11 patients (13.6%) in the 7-mg group and 10 patients (15.2%) in the 14-mg group had increased supine systolic blood pressure (≥160 mmHg and increase from baseline ≥20 mmHg), while three patients in each treatment group (who had normal blood pressure at baseline) had increased supine diastolic blood pressure (≥110 mmHg and increase from baseline ≥10 mmHg).

#### Hypersensitivity reactions

Over the entire treatment period, 22.2% of patients receiving 7 mg and 16.7% of those receiving 14 mg teriflunomide experienced non-serious transitory rashes; none led to treatment discontinuation, and no serious anaphylactic reactions were reported.

#### Malignancies

There was no specific pattern of occurrence with respect to the five cases of malignancies reported, and all were reported in the 7-mg group; basal cell carcinoma (*n*=1), renal cell carcinoma (*n*=2) and breast cancer (*n*=2). No haemato-oncological cancers (i.e. no leukemia (acute or chronic) or lymphoproliferative tumours) or liver cancers were reported. The exposure-adjusted incidence rate across both teriflunomide groups was 0.6 per 100 patient-years. The time to onset ranged from 2.3 years to 6.1 years.

#### Pregnancies

Six pregnancies, all in the 7-mg group, were documented during the course of the study. While four patients elected to terminate their pregnancy, two patients permanently discontinued treatment as soon as they were aware of their pregnancy and underwent a rapid drug elimination procedure with cholestyramine. Each gave birth to a healthy newborn with no structural defects or functional problems reported to date.

### Efficacy outcomes

#### Clinical efficacy

**Figure 2.**
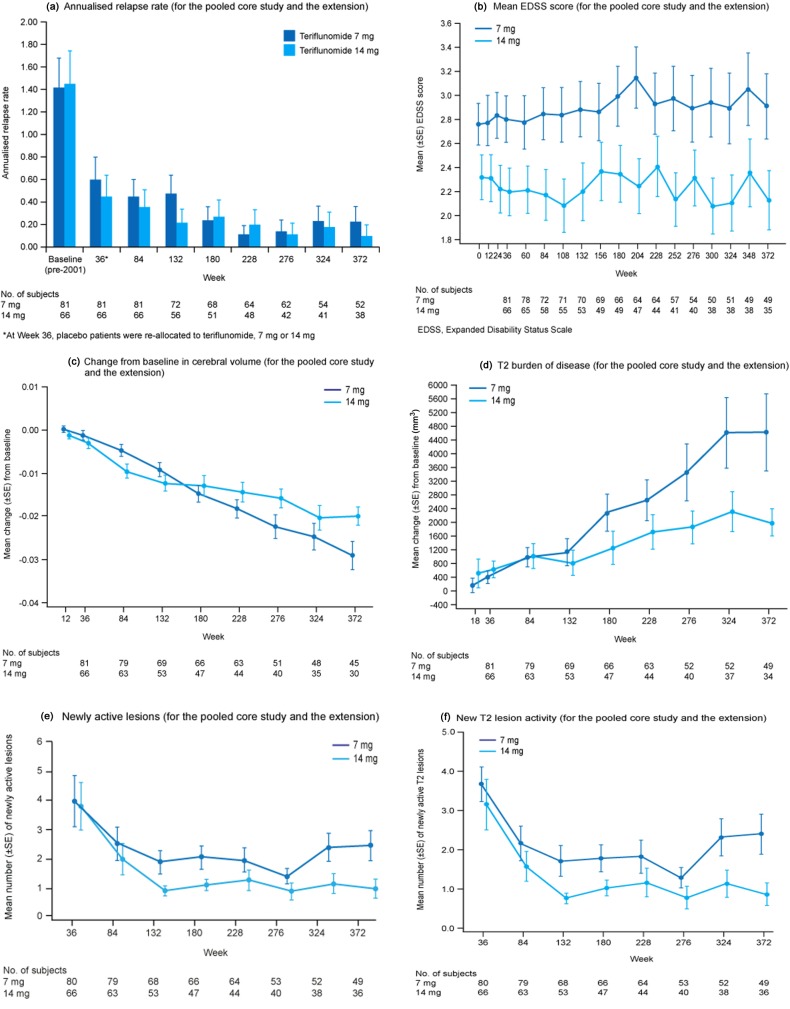
(a) Annualised relapse rate (for the pooled core study and the extension); (b) mean EDSS score (for the pooled core study and the extension); (c) change from baseline in cerebral volume (for the pooled core study and the extension); (d) T2 burden of disease (for the pooled core study and the extension); (e) newly active lesions (for the pooled core study and the extension) and (f) new T2 lesion activity (for the pooled core study and the extension).

ARRs decreased over the 372-week evaluation period in both teriflunomide groups (Table 3 and Figure 2(a)), with a trend towards a lower ARR in the 14-mg group at Week 372. Overall, patients had minimal disability progression (measured by EDSS scores) throughout the study period (Table 3 and Figure 2(b)). EDSS scores were higher in the 7-mg group at baseline, and these differences remained throughout the study.

**Table 3.** Magnetic resonance imaging (MRI) and clinical outcome measures (for the core study and the extension).

	Teriflunomide 7 mg			Teriflunomide 14 mg		
	Placebo/7 mg (*N*=29)	7 mg/7 mg (*N*=52)	Combined (*N*=81)	Placebo/14 mg (*N*=26)	14 mg/14 mg (*N*=40)	Combined (*N*=66)
**Relapse**						
Number of patients with ≥1 relapse, *n* (%)						
With relapse	17 (58.6)	30 (57.7)	47 (58.0)	11 (42.3)	18 (45.0)	29 (43.9)
Without relapse	12 (41.4)	22 (42.3)	34 (42.0)	15 (57.7)	22 (55.0)	37 (56.1)
Number of relapses, *n* (%)						
1	9 (31.0)	7 (13.5)	16 (19.8)	4 (15.4)	11 (27.5)	15 (22.7)
2	2 (6.9)	10 (19.2)	12 (14.8)	3 (11.5)	0	3 (4.5)
3	3 (10.3)	7 (13.5)	10 (12.3)	2 (7.7)	6 (15.0)	8 (12.1)
≥4	3 (10.3)	6 (11.5)	9 (11.1)	2 (7.7)	1 (2.5)	3 (4.5)
Total number of relapses	36	80	116	26	36	62
Total patient-years followed	161.1	270.4	431.5	122.1	199.0	321.1
Annualised relapse rate^a^	0.224	0.296	0.279	0.213	0.181	0.200
**EDSS (Week 372)**						
Mean (SD)	3.39 (1.64)	2.60 (2.03)	2.91 (1.91)	1.58 (1.16)	2.41 (1.55)	2.13 (1.47)
Median (min:max)	3.00 (1.5:6.5)	2.25 (0.0:7.0)	2.50 (0.0:7.0)	1.75 (0.0:3.5)	2.50 (0.0:6.5)	2.00 (0.0:6.5)
Mean (SD) change from baseline	0.97 (1.07)	0.23 (1.35)	0.52 (1.29)	–0.17 (1.25)	0.61 (1.11)	0.34 (1.20)
Median (min:max) change from baseline	0.50 (–0.5:3.0)	0.00 (–2.0:4.0)	0.50 (–2.0:4.0)	0.00 (–2.5:1.5)	0.50 (–1.5:3.0)	0.00 (–2.5:3.0)
**MRI (Week 372)**						
**T2 burden of disease (mm^3^)**						
Mean (SD) change from baseline	5714.79 (9740.56)	3846.58 (6415.27)	4609.11 (7900.90)	2039.98 (2649.14)	1969.68 (2203.36)	1994.49 (2330.22)
Median (min:max) change from baseline	2013.60 (–2552.3:38354.9)	1295.80 (–5178.3:27266.9)	1567.20 (–5178.3:38354.9)	1021.90 (–242.3:7867.5)	1191.85 (131.7:8965.8)	1191.85 (–242.3: 8965.8)
Mean (SD) percentage change from baseline	61.04 (85.65)	63.77 (85.78)	62.66 (84.84)	39.34 (53.88)	90.26 (113.85)	72.28 (99.13)
Median (min:max) percentage change from baseline	38.67 (–26.7:338.7)	41.39 (–14.0:397.8)	40.14 (–26.7:397.8)	16.54 (–29.6:153.5)	49.85 (1.8:436.0)	35.99 (–29.6:436.0)
**Cerebral volume**						
Mean (SD) change from baseline	–0.0332 (0.0280)	–0.0263 (0.0174)	–0.0290 (0.0222)	–0.0181 (0.0153)	–0.0207 (0.0102)	–0.0199 (0.0118)
Median (min:max) change from baseline	–0.0350 (–0.080:0.016)	–0.0230 (–0.072:0.002)	–0.0240 (–0.080:0.016)	–0.0130 (–0.043:0.001)	–0.0230 (0.040:0.000)	–0.0225 (–0.043:0.001)
Mean (SD) percentage change from baseline	–4.19 (3.58)	–3.36 (2.29)	–3.69 (2.87)	–2.29 (2.00)	–2.60 (1.28)	–2.51 (1.50)
Median (min:max) percentage change from baseline	–4.36 (–10.5:2.0)	–2.90 (–9.9:0.2)	–2.92 (–10.5:2.0)	–1.55 (–5.8:0.1)	–2.67 (–5.0:0.0)	–2.6 (–5.8:0.1)
**New T2 lesions**						
Mean (SD) number	1.40 (2.30)	1.97 (3.21)	1.73 (2.86)	0.33 (0.78)	0.83 (1.76)	0.67 (1.51)
Median (min:max)	1.0 (0.0:8.0)	0.00 (0.0:13.0)	0.00 (0.0:13.0)	0.00 (0.0:2.0)	0.00 (0.0:6.0)	0.00 (0.0:6.0)
**Newly enlarging T2 lesions**						
Mean (SD) number	0.50 (0.89)	0.76 (1.38)	0.65 (1.20)	0.33 (0.49)	0.13 (0.45)	0.19 (0.47)
Median (min:max)	0.00 (0.0:3.0)	0.00 (0.0:5.0)	0.00 (0.0:5.0)	0.00 (0.0:1.0)	0.00 (0.0:2.0)	0.00 (0.0:2.0)
**Newly active T2 lesions^b^**						
Mean (SD) number	1.90 (2.83)	2.72 (4.05)	2.39 (3.59)	0.67 (0.98)	0.96 (2.03)	0.86 (1.74)
Median (min:max)	1.0 (0.0:10.0)	1.00 (0.0:14.0)	1.00 (0.0:14.0)	0.00 (0.0:3.0)	0.00 (0.0:8.0)	0.00 (0.0:8.0)
**T1 Gd-enhancing lesions**						
Mean (SD) number	0.50 (1.15)	0.41 (1.05)	0.45 (1.08)	0.00 (0.00)	0.42 (1.47)	0.28 (1.21)
Median (min:max)	0.00 (0.0:4.0)	0.00 (0.0:5.0)	0.00 (0.0:5.0)	0.00 (0.0:0.0)	0.00 (0.0:7.0)	0.00 (0.0:7.0)
**Newly active lesions**						
Mean (SD) number	1.90 (2.83)	2.79 (4.14)	2.43 (3.65)	0.67 (0.98)	1.08 (2.36)	0.94 (2.00)
Median (min:max)	1.00 (0.0:10.0)	1.00 (0.0:14.0)	1.00 (0.0:14.0)	0.00 (0.0:3.0)	0.00 (0.0:8.0)	0.00 (0.0:8.0)

The baseline refers to the start of the placebo-controlled study.

a The total number of relapses that occurred during the treatment divided by the total number of patient-years treated in the study.

b New and newly enlarging T2 lesions.

#### MRI outcomes

There was a smaller reduction in cerebral volume (percentage change from baseline) in the 14-mg groups compared with the 7-mg groups, with a trend for the greatest loss of cerebral volume in the placebo/7-mg group (Table 3 and Figure 2(c)). There was also a smaller increase in T2 BOD (percentage change from baseline) in the 14-mg groups compared with the 7-mg groups at Week 372, with a trend for the greatest increase occurring in the placebo/ 7-mg group (Table 3 and Figure 2(d)). New MRI lesion activity (new and newly enlarging T2 lesions, and newly active lesions) was higher in the placebo groups compared with the teriflunomide treatment groups at the end of the core study. This activity was reduced with teriflunomide treatment during the extension, where the number of newly active lesions appeared lower in the 14-mg groups compared with the 7-mg groups (Table 3 and Figure 2(e)); a similar pattern was observed for newly active T2 lesions (Table 3 and Figure 2(f)).

#### Quality of life

Over the entire study period, there was a mean increase of 3.8 points on the patient-rated FIS scale, which was not considered to be clinically relevant (effect size=0.12 at Week 372 on the total FIS, which has a maximum score of 160). Patients had a mean reduction of 2.0 on the MSQoL-54 at Week 372, with negligible effect size (0.11) on the mental health composites and a decrease of 6.1 points with small effect size (0.35) on the physical health composites.

## Discussion

Oral teriflunomide, 7 mg and 14 mg, was well tolerated in patients with RMS with up to 8.5 years of exposure. Most TEAEs reported were mild to moderate, with only isolated cases of SAEs. TEAEs observed during this long-term study were similar in nature to those reported in the shorter-term teriflunomide phase 2 and phase 3 (TEMSO) placebo-controlled clinical trials.^9,10^

No serious opportunistic infections or hypersensitivity reactions were reported and there were only isolated reports of WBC decreases, none of which led to treatment discontinuation.

The incidence rate of malignancies in the study population, which comprised mostly Canadian patients, was within the range expected for the Canadian population at large.^12^ The cases reported were not organ-specific, there was no apparent dose-effect, and a variety of different types of cancers were reported, with no pattern reminiscent of that observed in immunocompromised patients or in patients with Epstein-Barr virus-related lymphomas, human herpesvirus-8-related Kaposi’s sarcoma, HPV-related ano-genital cancers, or Merkel cell polyomavirus-related cancers. Furthermore, there were no haematological, lymphoproliferative, liver cancers, or lymphomas reported.

Two patients who became pregnant during the study decided to continue with their pregnancy; they permanently discontinued treatment and underwent a rapid drug elimination procedure with cholestyramine. Both patients gave birth to healthy babies, and no structural defects or functional problems have been reported to date. Since teriflunomide has a long elimination half-life, the option of a procedure to rapidly eliminate teriflunomide from the body is reassuring, particularly for patients who are or are planning to become pregnant, and in situations of possible overdose.^11^

Disability progression, ARRs and MRI activity remained low throughout the course of the extension, providing evidence that the previously reported beneficial effects of teriflunomide on clinical and MRI endpoints are maintained over the long-term, for up to 8.5 years. There was a trend towards a dose-dependent benefit with teriflunomide 14 mg on several MRI parameters (including T2 BOD, cerebral volume, new and newly enlarging T2 lesions and newly active lesions), which is also consistent with that observed in previous teriflunomide clinical trials.^9,10^ Patient-reported fatigue and mental health-related quality of life (HRQoL) remained stable over the course of the long-term extension study, and the physical decrease in HRQoL paralleled the benefits observed on EDSS scores.^13^

Although findings are limited by the relatively small number of patients who remained on treatment at the interim cut-off date (85/179 patients), this study provides useful data which will help clinicians understand the safety profile of teriflunomide over the long-term. To the best of our knowledge, this study of teriflunomide in RMS provides the longest exposure data of any oral DMT to date.
